# COVID‐19‐another influential event impacts on laboratory medicine management

**DOI:** 10.1002/jcla.23804

**Published:** 2021-05-25

**Authors:** YunTao Luo, JingHua Wang, MinMin Zhang, QingZhong Wang, Rong Chen, XueLiang Wang, HuaLiang Wang

**Affiliations:** ^1^ Shanghai center for clinical laboratory Shanghai China

**Keywords:** COVID‐19, laboratory medicine, public health emergency, quality management, rapid‐launched LDTs

## Abstract

**Background:**

Before public health emergencies became a major challenge worldwide, the scope of laboratory management was only related to developing, maintaining, improving, and sustaining the quality of accurate laboratory results for improved clinical outcomes. Indeed, quality management is an especially important aspect and has achieved great milestones during the development of clinical laboratories.

**Current status:**

However, since the coronavirus disease 2019 (COVID‐19) pandemic continues to be a threat worldwide, previous management mode inside the separate laboratory could not cater to the demand of the COVID‐19 public health emergency. Among emerging new issues, the prominent challenges during the period of COVID‐19 pandemic are rapid‐launched laboratory‐developed tests (LDTs) for urgent clinical application, rapid expansion of testing capabilities, laboratory medicine resources, and personnel shortages. These related issues are now impacting on clinical laboratory and need to be effectively addressed.

**Conclusion:**

Different from traditional views of laboratory medicine management that focus on separate laboratories, present clinical laboratory management must be multidimensional mode which should consider consolidation of the efficient network of regional clinical laboratories and reasonable planning of laboratories resources from the view of overall strategy. Based on relevant research and our experience, in this review, we retrospect the history trajectory of laboratory medicine management, and also, we provide existing and other feasible recommended management strategies for laboratory medicine in future.

## Graphical abstract

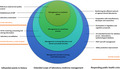

## 1 INTRODUCTION

Before the coronavirus disease 2019 (COVID‐19) pandemic, laboratory medicine management activities mainly remain inside of the laboratory, including reagent and instrument maintenance, daily quality assessment, errors elimination, turnaround time reduction, etc. The development process is briefly divided into several distinct phases, including standardization of biological reference material,^1^, ^2^ introduction of quality control,^3^, ^4^ establishment of standard operating procedures,^5^, ^6^ laboratory automation, and laboratory management accreditation.^7^, ^8^, ^9^ Based on history trajectory, we determined that the previous management goal of laboratory medicine only aimed to develop, maintain and improve the quality of accurate laboratory results.

However, since the COVID‐19 outbreak, the laboratory management system has become particularly vulnerable. The most prominent issue is that tremendous burden is placed on clinical laboratory resources. Generally, because of cost containment strategies and laboratory space size, clinical laboratories have been designed and organized to sustain a customized volume of tests for local health system,^10^ rather than concern rapidly expand their testing capabilities. Therefore, existing laboratory management system is implemented in a planned way under this context and related laboratory material and personnel resources are deployed in separate laboratories, less considered the time efficiency for rapid‐launched laboratory‐developed tests (LDTs) and the efficient network of regional clinical laboratories. Unfortunately, with COVID‐19 continues to spread, the daily activity in separate clinical laboratories is rapidly saturated or even overwhelmed and disrupted by the large numbers of tests for COVID‐19.^10^ Routine management inside the separate laboratories could not cater to the demand of the COVID‐19 public health emergency. Among emerging issues, the prominent challenges are undoubtedly the rapid expansion demand of testing capabilities and rapid‐launched LDTs for urgent clinical application. Rapid expansion of testing capabilities also leads to laboratory medicine resources and personnel shortages; the activity of rapid‐launched LDTs appeared to contradict the previous LDTs management mode that needs sufficient validation in large‐scale clinical practice, posing dilemmas for laboratory management.

Besides personnel and material resources, huge amount of information processing and large database management also become more critical during COVID‐19 pandemic.^11^, ^12^ Recent computer science technology in laboratory medicine is more than just simple laboratory information system (LIS) as before. Development of artificial intelligence (AI) technology in medical laboratory is necessarily trend and could actually be addressed some current management problems, especially in decreasing the burden on the healthcare system and rational resource allocation.^12^, ^13^, ^14^ Some experts indeed provide automated intelligent frameworks which show great potential benefits in many aspects, such as laboratory diagnosis, laboratory treatment, epidemiological investigation, and biological data mining for fighting COVID‐19.^13^, ^14^, ^15^, ^16^, ^17^ However, due to some technical aspects (inappropriate analysis methods, limitations of utilizing data mining and machine learning algorithms, low accuracy and computational efficiency),^12^, ^15^ such applications still remain insufficient given this worldwide crisis posed by COVID‐19 to global public health. Proper and effective handling of these problems facilitates appropriate transformation for medical laboratories. In this review, we introduce the brief history trajectory of laboratory medicine management and also, from the view of overall strategy, provide existing and potential feasible management strategies for laboratory medicine in future.

## 2 HISTORY OF LABORATORY MEDICINE MANAGEMENT

**FIGURE 1:**
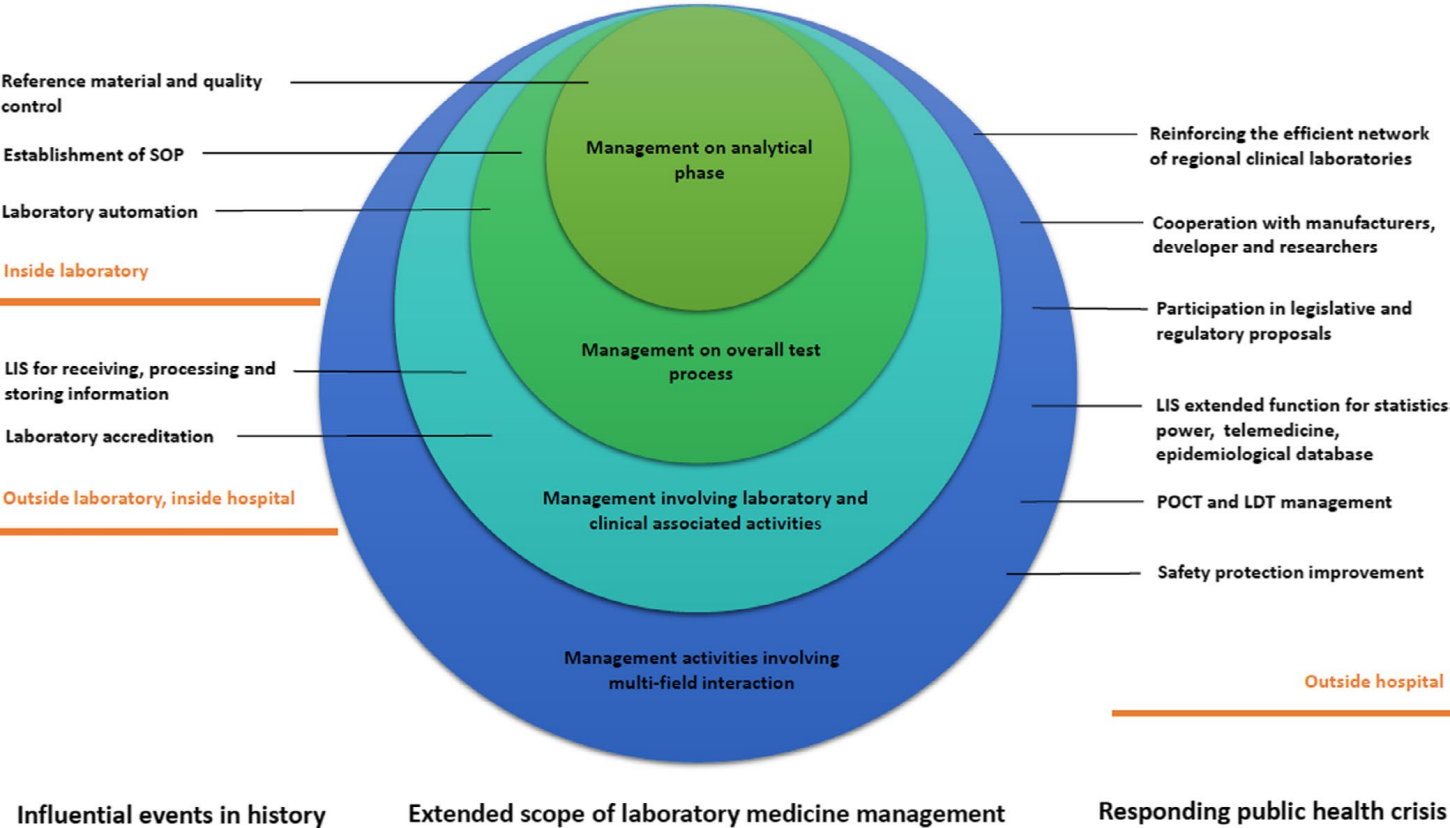
Influential events during the history impacted laboratory management activities (left). After COVID‐19 outbreak, the scope of management outside the laboratory has extended exponentially the scope (right).

In the past, influential events impacted laboratory medicine as well as advancements in the management development (Table 1). The development process can be summarized in two aspects, including the scope of laboratory management and the application of advanced technology (Figure 1).

**TABLE 1:** Milestone events for laboratory medicine management.

Time	Event	Ref
1921	The first international biological standardization meeting in London	^1^
1924,1950	The first control chart described by Shewhart in 1924 and a similar concept was first introduced into clinical chemistry by Levey and Jennings in 1950	4, 18
During the 1960s	The development of quality management in medical laboratory testing started by Norwalk Hospital	^5^
1961	The CAP founded its accreditation program	^23^
1965	The first meeting concerning “quality healthcare movement” convened in Chicago	^63^
1970s	LIS began to be applied in the clinic	64, 65
1980s	Automation in the clinical laboratory	^9^
Since the 1990s	A remarkable transformation of clinical laboratory management made in Finland, Ireland, Netherlands, Sweden, Switzerland, and the UK,implementing ISO 15189 and ISO 22870	20, 21
1990s	Application of PCR in medical laboratories	^26^
1997	NACB promulgated its first SOP	^66^
2003	ISO 15189 standard, medical laboratory requirements for quality and competence, first officially published	^23^
2004	A five‐phase examination process model was proposed, referred to as “filter model” or “NEXUS vision”, covering a wider scope of laboratory medicine	5, 67
2007; 2012	The ISO 15189 standard, revised in 2007 and again in 2012.	^23^
Since 2005	ISO 15189 was introduced in medical laboratories with rapid growing international adoption	^23^
2002–2020	Serious infectious diseases events (SARS, MERS, SARS‐CoV−2, etc.)impacted laboratory medicine management	61, 68

The scope of laboratory management is increasing compared to previously. Initial important milestones were established as reference measurement system (first biological standardization meeting; 1921^1^). In addition, the introduction of quality control (by Levey and Jennings; 1950 ^3^, ^18^) transformed the practice of quality management in the analytical phase. With time, scholars realized errors in clinical laboratory tests did not only occur in the analytical phase ^19^ and thus a wider scope of quality management was required. Peer‐consensus SOPs were previously established and implemented into medical laboratories, which would ensure quality during the pre‐analytical and post‐analytical phases.^5^ During the 1990–2000s, more researchers and related personnel emphasized the importance of laboratory quality management systems (QMS). Laboratory accreditation became an essential aspect of laboratory medicine management and swept worldwide.^20^, ^21^ The most unique QMS for medical laboratories is ISO 15189, which obtained rapid international adoption in the 2000s.^8^ Laboratory accreditation further strengthened the goal for laboratory management and extended the scope not only for improving the quality of the entire analytical phase but also offered an overarching management structure, including personnel safety and customer satisfaction.^22^, ^23^, ^24^

Another aspect impacting management development of laboratory medicine is the application of advanced technology. LIS and laboratory automation were introduced into medical laboratories. Advanced automated analyzers allow for increased workload capacity and reduce personnel resources, increasing skilled manpower focusing on value‐added activities.^25^ LIS is a connecting platform that facilitates automatic data transfer, leading efficient management in the communication between laboratory and clinicians. Other influential technology that impacts laboratory medicine includes the polymerase chain reaction (PCR) that was developed in the 1990s.^26^ It comprehensively revolutionized diagnostic techniques and contributed to the rapid development of LDTs for nucleic‐acid‐based methods. It also greatly transformed general legislative and regulatory proposals regarding LDTs.

## 3 EXTENDED SCOPE AND RESPONSIBILITIES OF LABORATORY MANAGEMENT AFTER COVID‐19

Due to the increase in advanced technology applications and other influential public health emergencies impacting the laboratory medicine discipline, the management of laboratory medicine faced many challenges (Figure 1). Previous laboratory management models may not have adapted to the new requirements outside of laboratory, including LDTs and POCT management, the efficient network of regional clinical laboratories and laboratories resources management, information processing and database management, etc.

### 3.1 Rapid‐launched LDTs for clinical application

#### 3.1.1 Conditions for rapid LDTs

Since PCR was generated in the 1990s, a variety of LDTs were established and launched in clinical laboratories. Management activities of laboratory medicine were extended to interact with manufacturers, developers, and researchers,^10^, ^27^ as well as involving legislative and regulatory proposals.^28^, ^29^

In general, an LDT may need validation in large‐scale clinical practice. It takes time to develop routine clinical laboratory testing. Furthermore, to effectively acquire an available license, the development of LDTs generally tends to operate commercially rather than in hospital laboratories.^28^, ^29^ However, during the COVID‐19 pandemic, many countries launched heterogeneous LDTs for COVID‐19 detection ^27^ and clinical laboratories contributed support to associate with manufacturers and other research institutions.^10^, ^27^ In China, at least 11 nucleic‐acid‐based methods and 8 antibody detection kits were approved for the clinic.^30^ Also, other emerging LDTs for detecting COVID‐19 were generated. Selecting appropriate LDTs for rapid clinical application was a new issue for medical laboratory management in urgent situations. Certain conditions need to be fulfilled for an LDT to be rapidly applied into medical laboratories. These conditions are as follows:

1. Similar technologies or testing have been developed and used in clinical laboratories.

For example, an RT‐LAMP test used for MERS‐CoV detection ^31^ previously showed poor analytical sensitivity. To improve this deficiency, assay readout signals were amplified by a variety of techniques such as thermal imaging and the assembly of multiple gold nanoparticles. Thus, during the COVID‐19 pandemic, the RT‐LAMP assay was easily redesigned by manufacturers and adapted rapidly to detect COVID‐19 RNA or proteins in the clinical laboratory.

2. Some LDTs in later stages may quickly be applied clinically.

Some scholars delineate diagnostic technology development into four phases, including conception/phase 1, clinical test/phase 2, clinical trial/phase 3, and commercialization/phase 4.^30^ LDTs in phases 1 and 2 cannot be used to immediately diagnose COVID‐19. These early stages typically occur in an academic setting or at the proof‐of‐concept stage and have little to do with clinical laboratories. In urgent situations, some LDTs in later stages, such as phase 3 advances to clinical trials with a large patient cohort or phase 4 that is commercialized and used in patients, have increased possibility in speeding up clinical application.

3. Effective evaluation of heterogeneous LDTs for selection.

Daily laboratory management mainly sustains the quality of routine laboratory results. However, since the recent rapid development of LDTs for COVID‐19, heterogeneous commercial reagent kits lack enough evidence for clinical validation. Before their use in clinical laboratories, a thorough analytical clinical assessment is still indispensable.^10^ Currently, the molecular technology approach is major clinical diagnostic strategy implemented in a majority of countries. Nevertheless, obvious features are among different diagnostic approaches or platforms. Based on studies conducted in China and other countries, the biggest issue is heterogeneous sensitivity and specificity among different approaches. For example, the first approach approved by WHO consists of three viral genes (E, RdRp, and N) as targets.^32^ A second approach, launched by the Centers for Disease Control and Prevention (CDC) in the United States, used a combined approach for the N1/2/3 gene using the RNase P gene as a control.^27^ Some research inferred that various gene targets with different criteria may lead to discrepancies in interpretation.^33^ After widespread testing of patient populations, data from clinical laboratories indeed confirmed heterogeneous false‐negative (or positive) test results among various testing protocols.^10^, ^27^ From our experience, when an LDT is prepared for clinical application, parallel testing with other reagent kits for assessment of sensitivity and specificity is critical. The selection of heterogeneous LDTs may come from direct experience (awareness of the strengths and limitations of different assays and testing platforms) as well as communication with developers, scientific literature, sharing of peer‐to‐peer information, and recent international conferences.

#### 3.1.2 Optimization of LDT protocols for high demand

Previously, LDTs always played a critical bridge role between the forefront of diagnostic innovation and translational medicine ^34^, ^35^ and were less considered as emergency strategies for a public health crisis. When prepared for clinical application, an LDT always contained small‐scale test volumes in reference laboratories or other qualified hospital laboratories. With small‐scale test volume and sufficient related laboratory facilities, experienced staff in reference laboratories can operate experimental procedures strictly depending on protocols. Since the SARS‐CoV‐2 outbreak, the situation for LDTs is changed. The abundant demand for COVID‐19 detection was unprecedented since tests needed to be performed in large quantities routinely. Few reference laboratories were able to afford this unexpected burden.^10^ Therefore, more community hospitals in peripheral centers were assigned high sample throughput for COVID‐19 detection. A large number of hospital laboratories lack corresponding PCR infrastructure.^30^ More importantly, they also lack corresponding SOPs ensuring quality of the entire analytical phase. Generally, protocols developed by manufacturers are only suited for manufacturing‐oriented QSRs.^28^ Thus, LDT protocols cannot be directly introduced into clinical laboratories for ample routine test volumes (Table 2). Before implementing COVID‐19 detection LDTs, modifications and optimization of protocols in order to meet hospital laboratory requirements is indispensable.^28^ The foremost prerequisite is to understand potential pre‐analytical and analytical vulnerabilities in laboratory diagnosis.^33^ From our experience, continual modifications in the procedures for COVID‐19 detection need to be performed due to the constant update of knowledge, regulations, and legislations. Such modifications are performed in the pre‐analytical phase regarding COVID‐19 specimen stability, specimen type, and different population screenings. These modifications were also performed in the post‐analytical phase and included an appropriate cutoff value and interpretation of results.^27^, ^36^, ^37^ It is worth mentioning that an innovative pooling protocol was proposed by some laboratories for larger population screenings. As more widespread testing for COVID‐19 detection in the general population and asymptomatic individuals is needed, a large shortage of testing supplies is inevitable. To further conserve resources using existing testing methodologies, some laboratories have investigated pooling many patient samples to decrease the number of tests.^38^ They have modeled patient pooling and proposed algorithms that optimize positive sample detection and testing efficiency.^38^ Proposed pooling protocols are not a normal operation process in routine tests and may result in some disadvantages, such as a slight loss in sensitivity from diluting positive samples with negative samples. However, optimization of protocols can aid clinical laboratories during the worst period of the COVID‐19 pandemic.

**TABLE 2:** Differences between protocols and SOPs.

	Protocol	SOP
Terminology usage	Generic use or exclusive abbreviation	Peer‐consensus standard
Purpose	Aim for individual experiment design under specific application conditions	Ensures the quality of all analytical phases
Document formation	Separate file	Composed by one or more relevant protocols or other controlled documents
Applicability	Scientific medical research or unaccredited tests	Clinical routine or accredited tests
Files compiler	Manufacturer, designers, and developers	Clinical laboratory managers and peer experts
Revision procedures	Depend on each developer for individual experiments	If revisions are required, re‐authorization is needed
Description scope	Often described for specific operation step sonly	Detailed normative description for the entire procedure
Personnel requirements	Professionals or scientists	Clinical laboratory staff or peers
Feasibility of implementation	Options or references	Mandatory implementation once approved

### 3.2 POCTs and LIS

Point‐of‐care devices (POCT) reduce turnaround time or even exempt unnecessary steps during the sample collection process.^39^, ^40^ Considering these advantages, most countries realized an urgent priority in launching and establishing proper POCTs for rapid COVID‐19 diagnosis.^30^, ^41^ Currently, related diagnostic assays and technologies have shown clinical feasibility for COVID‐19 POCTs, including microfluidic devices, lateral flow antigens, and serological tests.^27^, ^30^, ^42^, ^43^, ^44^

Nevertheless, compared with laboratory‐based testing, POCTs still face two major management issues. One issue is that current POCTs for COVID‐19 screening are often performed under various experimental conditions. Due to a shortage in laboratory staff and patients self‐quarantine, POCTs may frequently be performed by physicians, nurses, or even non‐professionals outside of the laboratory.^23^ Although the previous view is that POCTs with unique advantages of simple operation without needing professionally trained operators,^45^ the implementation of some POCTs for COVID‐19 still requires considerable professional knowledge and safety protection skills. For example, recent research reported that serological tests show a high frequency of cross‐reactivity,^30^, ^46^ and plasma samples may be infectious.^47^ Unqualified personnel may cause risks in the application of POCTs. As a result, sufficient personnel training, technical support, result comparability, and consultation become a requisite for POCT management outside of the laboratory. Another issue is epidemiological surveillance of COVID‐19 in POCT devices. Presently, many POCT devices lack bidirectional interfaces or have insufficient integration with LIS.^45^ A deficiency and neglect of data transmission may cause inaccurate epidemiological data from LIS statistics, leading to issues in controlling pandemics such as COVID‐19. Nevertheless, this situation may be improved by the implementation of smartphone‐based POCTs.^30^, ^45^ Previous reports demonstrated that smartphones are suitable to be incorporated into other promising technology, including paper‐based sensors, microfluidic chips, and flexible electronics.^48^ Since more patients with mild symptoms are sent home for self‐quarantine, demands for telemedicine and off‐site diagnosis are unprecedented. Data telecommunications for the POCT and LIS systems are becoming an important element. The extended function of smartphone‐based systems must possess connectivity, computational power, and hardware to facilitate electronic reporting and epidemiological databases.^30^

### 3.3 Laboratory medicine resources and personnel

During the COVID‐19 pandemic, hospital laboratories faced the need for an amplified volume of tests. Daily activities of clinical laboratories may be rapidly saturated or disrupted by an amplified volume of COVID‐19 screening tests. On one hand, automation prevalence indicates that clinical laboratories have more high‐throughput instrumentation, less employees, and public healthcare facilities. On the other hand, clinical laboratories, even those that were recently constructed, were designed to sustain a customized volume of tests for local hospital settings.^10^, ^49^ These facts considerably contributed to reduced flexibility in development responses. Existing laboratories may need to enhance their daily throughput, but this may limit their ability and may not be sustainable. Thus, effectively reshuffling laboratory medicine resources and recruiting urgent personnel are critical to face unexpected health crises.

In reshuffling laboratory medicine resources, a feasible solution is reinforcing the efficient network of regional clinical laboratories involving those not directly challenged by the outbreak. However, in turn, the frequent activities of regional clinical laboratories give rise to several unavoidable issues, such as infectious specimen transportation, biosafety requirements, and result consistency.^10^ Another effective alternative is to create new facilities within already existing buildings that can help perform tests in large volumes.^10^ New facilities should be constructed nearby medical sites, such as clinical wards, ICUs, and emergency departments, to fulfill minimum pre‐analytical quality requirements (especially sample transportation and collection).^50^ New facilities should be designed as mobile structures, such as trucks or caravans, as well as performed inside tents, shelters, or already constructed structures, such as sport stadiums, convention centers, and other public buildings.^47^ COVID‐19 detection tools in new facilities may introduce portable equipment or POCTs.^10^ The availability and extended use of these new facilities can be viewed as additional efficient laboratory medicine resources outside of centralized facilities during outbreaks and other biological hazards.^30^

To tackle the issue of personnel shortage, personnel can be recruited from other departments of laboratory medicine, healthcare fields, or as volunteers from other groups. Laboratory medicine staff may need to temporarily move to a new laboratory (eg, moving from a biochemistry to a virology laboratory) or may have to move to a new regional clinical laboratory.^10^ Since urgent personnel may lack direct experience, professional skills in virologic‐related assays, and awareness of biosafety protection, hands‐on training is required as soon as possible. Existing laboratory professionals may also provide a critical guide for urgent recruited personnel on‐site or online and define clear and temporary guidelines for their operations is an efficient manner.

### 3.4 Improvement of laboratory safety management

Presently, specimens for COVID‐19 detection collected from the upper respiratory tract or blood are often assigned to microbiological or molecular laboratories.^36^ These infectious disease related laboratories are traditionally categorized as having a high exposure risk. However, recent research already reported that SARS‐CoV‐2 could be isolated from the blood, feces, and urine of patients.^23^, ^47^, ^51^ These findings indicate that the virus can survive and is infectious through these specimens.^52^ Although data show the probability of contracting this virus by specimen contact is very low, it is still a potential risk. With the spread of COVID‐19 in many countries, numerous infected individuals are asymptomatic.^53^ Specimens from these individuals often involve other clinical laboratories, such as hematology or biochemistry laboratories, which are generally observed as low‐exposure risk departments ^27^, ^54^ and often lack essential personal protective equipment (PPE). With updated information regarding risk of viral spread, such safety awareness and security protection facilities in these departments will most likely improve.^10^, ^27^ Proper laboratory safety regulations will be revised and covered in a wider scope in low‐exposure risk laboratories.^55^

### 3.5 “Big data” management in laboratory and policy‐making

Presently, laboratory data management may face more advanced requirements, especially on epidemic disease investigation and surveillance. Rather than merely maintenance LIS data inside separate laboratories as usual, current big data survey need sufficient sharing data from wider regional clinical laboratories. For example, during the period of COVID‐19 pandemic, big data comes from two aspects (molecular‐based testing and routine testing).^56^, ^57^, ^58^ Data from molecular‐based testing such as RT‐PCR were considered vital for COVID‐19 verification of the course of infection at first and showed high sensitivity and specificity (78.2% and 98.8%) in some separate laboratories.^59^ Many countries attempted to rapidly expand their testing capabilities for COVID‐19 diagnosis and screening. However, with more data were available from wider regional laboratories, it was revealed that RT‐PCR test for COVID‐19 has moderate sensitivity (63–78%) and pharyngeal swabs seem to have the lowest sensitivity.^52^, ^59^, ^60^ Based on these results, strategy of laboratory management may also alter distinctively in different areas. In China currently, due to few cases of COVID‐19, increased testing for COVID‐19 screening are still considered as an effective way for epidemiological survey and control. The government and laboratory organizations also, therefore, modified the policy correspondingly. They require medical laboratories to use appropriate test kits with higher sensitivity (500 copies/ml or even 200 copies/ml) for aggressive early testing and recommend nasopharyngeal testing over oropharyngeal testing. Whereas in cities already being devastated by COVID‐19, some scholars consider that RT‐PCR test for COVID‐19 may not be advocated as a reliable surrogate for massive mild illness.^27^, ^47^ They argued that the large numbers of RT‐PCR testing with mild illness will have minimal effect on epidemic control and waste massive laboratory resources.^59^ They are more inclined to reduce testing of patients with mild disease, which could save testing materials so that sicker patients and healthcare professionals will have access to testing. Compare with molecular‐based testing, data from routine tests may also provide crucial support for epidemic disease surveillance. Multicenter, cross‐sectional studies have highlighted abnormal results of hematologic and biochemistry parameters from patients with COVID‐19,^10^, ^56^ related to disease severity and complications, and even showed geographic variability in COVID‐19 patients.^27^, ^32^, ^61^, ^62^ Therefore, effectively obtaining “big data” from routine testing may be a simple and cost‐effective approach for patient monitoring,^36^ epidemic disease investigation, and surveillance.^10^

However, how to effectively access and analyze “big data” from wider regional laboratories without additional burden on the laboratory is an unprecedented challenge for laboratory management. Fortunately, with recent progress in digitized data acquisition and machine learning methods, AI as another computing science technology is gradually changing medical laboratory practice, especially in huge of information processing and big data management.^14^, ^17^ A few papers have already reported their successful cases on convalescent‐plasma (CP) transfusion.^16^ In order to select the best CP for the most critical patients with COVID‐19, via machine learning and different analysis procedures, several intelligence rescue frameworks were designed.^15^, ^16^ AI techniques could handle huge of information from multicenter clinical database and solve multicriteria decision‐making for optimal match of donors/patients plasma, including classifying blood types, serological/protein biomarker criteria, and rational hospital distribution.^15^ Despite the relatively low number of studies in this field, but it can really contribute to make a difference in large database management in future.

In a word, qualified epidemic disease investigation and survey are critical for policymaking and laboratory resources redistribution. In addition to provide big data from wider regional clinical laboratories timely and accurate, the prerequisite of beneficial laboratory management strategies should also be fully combined with the situation and conditions of local regions.

## 4 OPEN ISSUES AND INNOVATIVE KEY SOLUTIONS

Here, we summarize related issues surrounding laboratory medicine management. Some solutions may not be the only option but can be used as an alternative solution. It is worth mentioning that different solutions also bring new unexplored problems which make laboratory management involved in other multidimensional fields (Table 3).

**TABLE 3:** Issues surrounding laboratory medicine management and key solutions.

Issues	Current status	Key solutions	New management problem
Rapid‐launched LDTs	Aim to optimal experimental conditions and sufficient clinical practice (time‐consuming)	Achieve the minimum clinical application requirements in equipment, personnel, SOP	Consensus on minimum clinical application requirements; reliable performance indicators definition (qualitative and quantitative)
Abundant testing demand on LDTs	Small‐scale test volumes in reference laboratories or few qualified hospital laboratories	Pooling samples to increase molecular testing throughput	Evaluate the sensitivity, specificity, reproducibility and verify optimal pooling approach ^69^ ; Define distinctive screening strategy in different populations and areas
POCT	Large‐scale instruments not applicable; Non‐professionals involved	Mobile biosafety laboratories ^70^	Instrument quake‐proof in mobile biosafety laboratories; operator body shape; hardware circuit debugging; software debugging; daily maintenance
LIS	Data outside laboratory disconnection with LIS (manual recording or miss data)	Smartphone‐based systems; intelligent connected devices	Remote data debugging; software settings
Big data survey	Survey data from regular regional meetings or publications	Real‐time data sharing from wider regional clinical laboratories; establishment of AI framework	Statistical software networking; survey data analysis; specific personnel training; regional organization and local government conduct and support; appropriate analysis methods for AI; limitations of machine learning algorithms
Laboratory material and personnel resources shortage	Resources and personnel in separate laboratory or department	Emergency resources reserve (acknowledge local healthcare plans, administrative duties, and political context); regional sharing and policy deployment; available staff from other groups, regional clinical laboratory or remote assistance work	Need accurate strategy and policy guidance; Identify staff regulations (eg, time on turn, recovery) under urgent situation; consensus on personnel training, SOP, report, and data interpretation

## 5 CONCLUSION

Although some academic literature recently mention related topics (laboratories findings, laboratories diagnosing tools, method sensitivity, and specificity for COVID‐19), they rarely discuss laboratory management from the view of overall strategy. Under current or future urgent public health situations, laboratory management must involve in multidimensional fields. For controlling outbreaks and epidemic disease surveillance, the important and essential management issues are rapid‐launched related LDTs or P2+ biosafety laboratories, which demand advanced requirements on existing quality management system. Besides traditional quality management (such as increasing workload demands, reducing errors and enhancing laboratory performance, etc.), time efficiency and reasonable laboratory resources reallocation are also major aspects of current laboratory management. In order to do the best, strategy and activities of laboratory management must be appropriate, involving consolidation of the efficient network of regional clinical laboratories, big data survey timely, accurate planning of laboratories resources and local political context, etc.

## AUTHOR CONTRIBUTIONS

YunTao Luo: Writing ‐ original draft; JingHua Wang; QingZhong Wang; Rong Chen; XueLiang Wang: Review and editing; MinMin Zhang: Editing and involving pre‐ or post‐publication stages; HuaLiang Wang: Conceptualization and review.
